# One-year Outcomes of Pachymetry and Epithelium Thicknesses after Accelerated (45 mW/cm^2^) Transepithelial Corneal Collagen Cross-linking for Keratoconus Patients

**DOI:** 10.1038/srep32692

**Published:** 2016-09-06

**Authors:** Xiaoyu Zhang, Ling Sun, Yingjun Chen, Meiyan Li, Mi Tian, Xingtao Zhou

**Affiliations:** 1 Eye and ENT Hospital of Fudan University, Myopia Key Laboratory of the Health Ministry, Shanghai, China

## Abstract

The thickness of corneal pachymetry and the epithelium after accelerated (45 mW/cm^2^) transepithelial corneal collagen cross-linking (CXL) for keratoconus were assessed in this prospective case series study. Twenty-eight patients were treated for keratoconus. The mean Kmax was 56.18 ± 7.90. The thinnest point, as assessed by optical coherence tomography (OCT), was 443.18 ± 39.75 μm. Accelerated transepithelial CXL was performed, and corrected distance visual acuity (CDVA), corneal topography, and OCT were recorded at 1 week postoperatively as well as at 1, 3, 6, and 12 months. The surgery was uneventful in all eyes. Postoperative epithelial edema was observed and faded in 3 days. The postoperative Kmax was 54.56 ± 8.81, 55.78 ± 8.11, 56.37 ± 8.71, 55.80 ± 7.92, and 55.47 ± 8.24 at 1 week, 1 month, 3 months, 6 months, and 12 months, respectively (all, P > 0.05). The thinnest postoperative corneal point, 439.04 ± 44.99 μm, was observed at 12 months (P = 0.109). The epithelial thickness decreased during the first postoperative week then showed a gradual recovery. Postoperative pachymetry thickness showed no significant changes for up to 12 months. Postoperative epithelial thickness decreased temporarily, then stabilized at month 12. Accelerated transepithelial CXL was shown to be effective and safe for the treatment of keratoconus.

Keratoconus (KC) is a degenerative, bilateral, asymmetrical, non-inflammatory disease that induces biomechanical corneal weakening due to aberrant changes in organization and the structure of stromal corneal collagen fibers^1^,^2^, which is a serious ocular disorder that can cause severe loss of vision^3^. The recommended treatment is deep anterior lamellar keratoplasty or a penetrating corneal graft for patients in advanced stages that have contact lens intolerance and/or dense stromal scars^4^.

Corneal collagen cross-linking (CXL) is used to treat keratoconus and keratectasia by strengthening corneal tissue through an interaction using a riboflavin photosensitizer and ultraviolet (UV) light. This treatment is based on an increase in covalent bonds within or between corneal collagen molecules that increases the biomechanical strength of the cornea^5^,^6^. This traditional protocol requires debridement of the corneal epithelium to promote diffusion of riboflavin into the corneal stroma^7^. Postoperative pain, infection, and even stromal haze are caused by the complex structural and physiological wound healing changes after CXL^4^,^8^. Long-term clinical studies showed the result of slowing, and in most cases blocking, keratoconus progression as well as improving refractive and topographic features^9^,^10^,^11^,^12^. Accelerated transepithelial or “epithelium-on” CXL is a technique performed without epithelial debridement by applying topical drugs to loosen the tight junctions of the corneal epithelial cells to facilitate riboflavin penetration through an intact epithelium. This technique was proposed to reduce the risk of complications caused by epithelial removal, such as postoperative pain and risk of infection, and ensure keratoconus stability as well^13^,^14^. This procedure uses various modified riboflavin formulations to facilitate diffusion through the corneal epithelium and leads to decreases in postoperative corneal thickness.

CXL is a surgical treatment that aims to stabilize the keratoconus, and transepithelial CXL offers the prospect of the presence of epithelium in visual rehabilitation. Early studies of this treatment showed that transepithelial CXL has the potential to offer patients both rehabilitation in visual acuity and corneal stability^12^. Observation of epithelial and corneal thickness changes after transepithelial CXL may reveal that the cornea has recovered after the damage caused by the riboflavin photosensitizer and ultraviolet (UV) light. The purpose of our study was therefore to evaluate the impact of accelerated (45 mW/cm^2^) transepithelial CXL on the changes in epithelial and corneal thickness in patients with progressive keratoconus by using spectral-domain anterior segment optical coherence tomography (OCT) to investigate the regeneration progress of the epithelium as well as the impact on different corneal sectors. We also compared the topographic and refractive results for validity and examined the endothelial cell density to evaluate safety.

## Patients and Methods

### Subjects

Patients diagnosed with progressive keratoconus were enrolled in a prospective case series study approved by the Ethics Committee of the Eye and ENT Hospital of Fudan University. The study adhered to the tenets of the Declaration of Helsinki and included 28 patients (28 eyes) treated for progressive keratoconus since December 2013. The mean age of the enrolled patients, which included 21 males and 7 females, was 23.61 ± 5.05 years ( ± SD) (Table 1). Written informed consent was obtained after each patient was informed about the nature and possible consequences of the study.

The inclusion criteria included axial corneal topography patterns consistent with keratoconus, elevated posterior surface elevation on topography mapping, an increase in central corneal astigmatism of at least 1.00D, an increase in the maximum cone apex curvature of at least 1.00D, a reduction of at least 10 μm in the thinnest point during the observation period, and biomicroscopic signs (e.g., Fleischer rings, Vogt’s striae, and Munson’s signs). Exclusion criteria included patients with a history of corneal surgery or chemical injury, corneal pachymetry less than 400 mm measured with a Pentacam (Oculus, Arlington, WA, USA), delayed epithelial healing, or pregnancy or lactation during the course of the study^15^.

### Accelerated cxl (kxl system)

**Figure 1:**
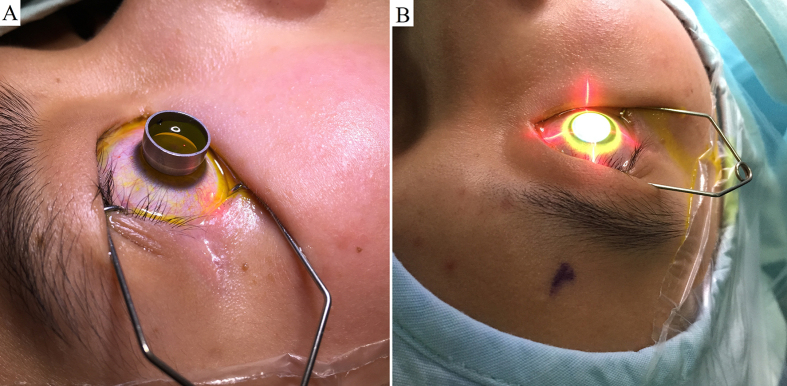
Photographs showing surgery being performed in patients with keratoconus. (**A**) applying sufficient Paracel in the corneal epithelial trephine for a total of 4 minutes, and applying sufficient VibeX Xtra in the corneal epithelial trephine for a total of 6 minutes. B, pulsed illumination at an interval of one second using 45 mW/cm^2^ for a surface dose of 7.2 J for a total of 5 minutes and 20 seconds.

The patients were placed in a supine position. Anesthetic eye drops were applied preoperatively, followed by use of a lid speculum. The corneal epithelium was left intact. Sufficient Paracel (Avedro, Waltham, MA, USA) containing 0.25% riboflavin-5-phosphate, hydroxypropyl methylcellulose, sodium edetate, trometamol, benzalkonium chloride, and NaCl in the corneal epithelial trephine (Model 52503B; 66 Vision-Tech, Suzhou, China) was used to completely cover the cornea for a total of 4 min. The cornea was then rinsed completely with VibeX Xtra (Avedro) containing 0.25% riboflavin-5-phosphate and NaCl, and sufficient VibeX Xtra was used in the corneal epithelial trephine for a total of 6 min. After using the epithelial trephine, the cornea was rinsed completely with balanced salt solution (BSS). Ultraviolet treatment was conducted using the KXL System (Avedro). The treatment protocol involved pulsed illumination for 1 s using 45 mW/cm^2^ for a surface dose of 7.2 J. The ultraviolet treatment procedure lasted for 5 min and 20 s (Fig. 1). The cornea was then rinsed completely with BSS, and a bandage contact lens was applied. Antibiotic drops were administered for 1 week, and topical steroids were applied for 16 days (four times a day initially, then reduced once every 4 days). Sodium carboxymethylcellulose drops were also prescribed.

### Ophthalmological examination

In the preoperative and postoperative examinations, the following parameters were assessed: corrected distance visual acuity (CDVA) (logMAR), intraocular pressure, manifest refraction, slit-lamp examination, corneal topography (measured with a Pentacam made by Oculus Optikgeräte, Wetzlar, Germany), endothelial cell density, and spectral-domain OCT (RTVue-100; Optovue, Fremont, CA, USA). The study participants were followed-up postoperatively at 1 week, and at 1, 3, 6, and 12 months.

### Data analysis and statistical evaluation

Results from the statistical analyses were expressed as the mean  ±  SD. For the missing data on a particular visit, the last observation carried forward (LOCF) method was used. A student’s *t* test was used to compare the postoperative changes from the preoperative values. Statistical analyses were performed using SPSS software, PASW 18.0 (SPSS, Chicago, IL, USA). Statistical significance was defined as a p-value < 0.05.

## Results

**Figure 2:**
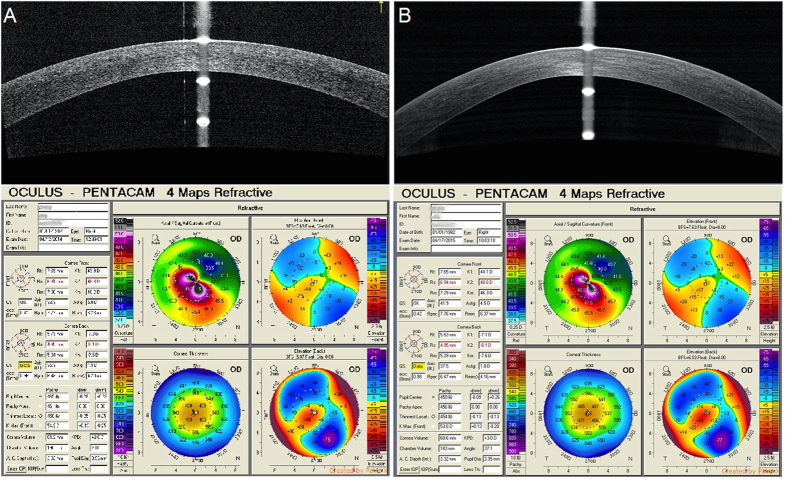
Relationship between the spectral-domain optical coherence tomography scan across the central 6-mm of the corneal apex in the vertical meridian and preoperative and 1-year postoperative Pentacam imaging (**A**,**B**).

All surgical procedures progressed successfully, and no intraoperative or postoperative complications occurred. No patients received additional surgery, such as penetrating keratoplasty (PKP), for any reason, and no haze or infection was observed. Among the 28 patients, nine received accelerated transepithelial CXL in both eyes, five received PKP in fellow eyes, seven lack follow-up data, and the other seven patients received no ophthalmic surgery in the fellow eyes. (Fig. 2).

### K value and central corneal thickness (cct)

The mean maximum apical keratometry value (Kmax) was 56.18 ± 7.90 preoperatively, and 54.56 ± 8.81, 55.78 ± 8.11, 56.37 ± 8.71, 55.80 ± 7.92, and 55.47 ± 8.24 postoperatively at 1 week, and at 1, 3, 6, and 12 months, respectively (P > 0.05). Corneal astigmatism was 2.64 ± 1.90 preoperatively and 2.74 ± 2.02 postoperatively at 12 months (P = 0.829). The CCT showed an increasing trend at postoperative 1 week and then returned to preoperative levels at postoperative 12 months. No significant changes were observed in the corneal posterior surface elevation (Table 2).

### Epithelial and corneal thicknesses

The mean epithelial sector thicknesses before and after treatment are listed in Table 3. The minimum number of epithelial points across the central 6-mm diameter zone changed from preoperative 41.68 ± 8.78 to postoperative 40.96 ± 10.27 at 12 months (P = 0.183), whereas the maximum number of epithelial points was 65.57 ± 7.10 and 65.26 ± 6.22, respectively (P = 0.656). Measurements at postoperative 1 week and at baseline values showed a decrease at all epithelial sector points (all, P < 0.001), and all sector epithelial points showed no significant change at postoperative 12 months compared with baseline values (all, P > 0.05).

The changes in the thickness of each corneal pachymetry sector are listed in Table 4. The average corneal thickness of most sectors did not show a significant change at postoperative 1 week compared with the baseline except for the paracenteral-superior, paracenteral-inferior, and mid-peripheral-inferior temporal sectors. At postoperative 3 months, all paracenteral pachymetry points were restored to baseline values (all, P > 0.05). There was no significant change in all sectors at postoperative 12 months compared with preoperative levels, except for the paracenteral-inferior and mid-peripheral-inferior temporal sectors.

### Visual acuity

At baseline, the uncorrected distance visual acuity (logMAR) was 1.02 ± 0.55, and significantly improved to 0.58 ± 0.34 at postoperative 12 months (P = 0.023). Preoperative CDVA (logMAR) was 0.31 ± 0.35 and postoperative CDVA at 12 months was 0.15 ± 0.19 (P = 0.056) (Table 2).

### Spherical equivalent and intraocular pressure (iop)

No statistically significant difference was found in the spherical equivalent (D) using subjective refraction at the postoperative follow-ups compared to preoperative values (all, P > 0.05). The spherical equivalent at baseline was −6.23 ± 3.81 D, and the spherical equivalents at postoperative 1 week, and at 1, 3, 6, and 12 months were −5.33 ± 3.36 D, −4.75 ± 2.60 D, −5.04 ± 2.97 D, −4.83 ± 2.9 D, and −5.35 ± 3.41 D, respectively. IOP was 12.29 ± 3.16 and 10.68 ± 3.72 mm Hg at baseline and postoperative 12 months, respectively, whereas endothelial cell density was 3264.67 ± 382.09 and 3303.92 ± 309.67, respectively, with no significant difference detected (P = 0.359, P = 0.621, respectively).

## Discussion

Treatments for ectatic corneal disorders, such as keratoconus, pellucid marginal degeneration, and iatrogenic corneal ectasia have changed dramatically in the past few decades. Previously, rigid contact lenses were the primary therapy. For eyes with contact lens intolerance, penetrating keratoplasty and implanting intrastromal corneal ring segments might be the next step in the treatment. A new treatment called corneal collagen cross-linking evolved and changed the perspective on corneal ectasia management^16^. A pilot clinical study was conducted in 2003 by Wollensak *et al*.^7^, which showed the first indication of the efficiency of this treatment. Accelerated CXL and transepithelial CXL were also reported as a potential alternative technique to treat keratoconus^17^,^18^.

Previous clinical and experimental studies have supported the efficacy and safety of the traditional CXL procedure for treatment of keratoconus^9^,^10^,^11^,^12^. To obtain the proper biomechanical effect of the traditional CXL procedure, the central corneal epithelium is debrided in an “epithelial-off” CXL procedure to reach a sufficient riboflavin concentration in the corneal stroma before inducing a photochemical reaction in the presence of ultraviolet-A (UVA) light to form free oxygen radicals. This procedure can unavoidably predispose patients to corneal infection and loss of corneal transparency due to abnormal corneal stromal scarring processes^19^.

In our study, patients underwent accelerated (45 mW/cm^2^) transepithelial CXL, which reduced the cross-linking time by increasing the UVA power, preserving the epithelium layer, and requiring significantly less time compared with traditional CXL as well as stabilizing the corneal thickness. This procedure facilitates use of CXL for patients with thin corneas or for less cooperative patients as well as increasing vision during the initial postoperative period and having fewer requirements for a sterile environment. To the best of our knowledge, this is one of the first studies describing the outcomes of accelerated (45 mW/cm^2^) transepithelial CXL as well as the postoperative corneal and epithelial thickness outcomes measured by OCT after accelerated transepithelial CXL using a pulsed UVA dose of 7.2 J/cm^2^ (intensity of 45 mW/cm^2^ lasts for 5 min and 20 s) to treat keratoconus.

In our study, we evaluated the safety of accelerated transepithelial cross-linking, and found that, compared with preoperative values, the postoperative 12-month endothelial cell density did not show a significant change, which indicates that the effect of increased UVA irradiance on endothelial cells was unremarkable. For patients with thin corneas, treatment security is difficult to ensure, and their endothelial cell density may be damaged. We therefore used accelerated transepithelial cross-linking, which preserved the epithelium, minimized UV damage, and had less impact on endothelial cells with greater treatment safety. However, the long-term safety of accelerated transepithelial cross-linking has yet to be studied.

In our study, accelerated transepithelial CXL showed clinical benefits with no changes in Kmax and no side effects, which indicates this procedure is stable and safe for treating patients with keratoconus. Relative to preoperative values, we found that patients with progressive keratoconus who received KXL showed long-term benefits from the procedure at their 12-month follow-up. The Kmax at postoperative 1 year was 55.47 ± 8.24D (P = 0.916), and corneal astigmatism changed from 2.64 ± 1.90D to 2.74 ± 2.02D (P = 0.829). The Pentacam and RTVue anterior segment OCT showed a decrease in the thinnest point of the cornea at the end of the follow-up period of 4.14 μm and 4.68 μm, respectively, which were not statistically significant compared to preoperative levels (P = 0.109, P = 0.376, respectively).

The traditional CXL procedure is shown to stabilize structurally weak corneas, halt keratoconus progression, and improve the visual acuity of affected patients^20^,^21^. The 10-year results after CXL treatment reported by Raiskup *et al*. included mean values for preoperative and postoperative maximum K values (53.2D and 49.56D, respectively) that showed a significant decrease over time with improved efficiency in treating progressive keratoconus and long-term stabilization of the cornea. Removing the corneal epithelium has been associated with high risks, such as infection^22^,^23^, stromal haze^24^, and other complications that affect the healing process^25^. Accelerated transepithelial CXL uses riboflavin solution containing chemicals that increase epithelial permeability to riboflavin and preserve corneal epithelium while localizing the reaction to the stroma. Benzalkonium chloride (BAC) containing ParaCel (Avedro) was reported to loosen the corneal epithelial junctions, which results in enhanced corneal permeability^26^,^27^. This procedure can be expected to achieve similar clinical results as the traditional CXL procedure with less damage as well as shorter operation times and a decrease in postoperative pain.

Using OCT preoperatively and postoperatively to determine corneal epithelial thickness, we found that due to the riboflavin invasive procedure, corneal epithelial thickness in surgical fields significantly declined at postoperative 1 week. All sectors of the corneal epithelium were restored to preoperative levels at 1 month postoperative, which was consistent with the corneal epithelial regeneration period. Recent studies reported that postoperative corneal thickness initially decreased then returned to baseline values at 3–6 months after the “epithelium-off” accelerated CXL procedure^28^,^29^. The recovery of corneal thickness in our study gradually returned the thickness to baseline values at postoperative 1 month, which was earlier than recovery times in previous studies. No demarcation line was observed through anterior segment OCT, which can be attributed to the epithelial permeability to riboflavin.

At postoperative 12 months, all epithelial sector points showed no significant changes compared to the baseline (P > 0.05). Regarding corneal thickness, we found significant decreases at postoperative 1 week in the paracenteral-superior and mid-peripheral-inferior temporal sectors as well as an increase in the paracenteral-inferior sector compared to baseline levels. We also found stable measurement results in all sectors at postoperative 12 months, except for the paracenteral-inferior and mid-peripheral-inferior temporal sectors, which indicates that the corneal epithelium and corneal thickness were stable in patients with progressive keratoconus who underwent accelerated transepithelial cross-linking. This outcome was consistent with the OCT findings and the patients’ descriptions. The mechanisms of corneal regeneration are unknown and should be addressed in future studies.

In our study, a corneal epithelial trephine was applied to increase epithelial permeability. Several options have been tried to improve the physicochemical results of CXL. Investigators reported that the significantly weaker biomechanical effect of “epithelium-on” CXL was possibly the result of insufficient and non-homogeneous transepithelial riboflavin diffusion into the corneal stroma^30^,^31^,^32^. Based on previous studies reporting increased epithelial riboflavin permeability by using hypotonic and isotonic solutions of riboflavin^13^,^33^, hypotonic riboflavin solution was used for our procedure. Furthermore, the KXL procedure was conducted with pulsed UVA light treatment, which has been shown to have a deeper apoptotic effect on the stromal layer compared to continuous treatment due to the induction of intraoperative oxygen reuptake^34^.

Keratoconus progression, as well as stability, generally does not follow a linear trend over time. Various studies have reported continuous flattening of the corneal curvature ranging from 0.72 to 2.2D for the first year^5^. However, at a 3-year follow-up, Goldich *et al*. found a reduction in Kmax to only 1.4D compared with 1.78D at 1 year, and 2.2D at 2 years^35^. It was suggested that pathological stromal remodeling might have contributed to the reversal of the initial flattening tendency. In other studies, patients showed stable keratometry values at postoperative 12 months, with progression in other years, and decreases from 14 to 19% to as much as 50% for pediatric patients^36^,^37^. Therefore, long-term corneal thickness measurements to assess stabilization effects are needed in future studies.

A limitation of our study involves the relatively low number of patients and lack of a control group undergoing standard CXL, which may increase the risk of bias. Additional trials (randomized controlled trials and larger sample size) are needed to better assess the impact of our procedure on keratoconus stabilization, especially for younger patients.

In conclusion, our results indicate accelerated (45 mW/cm^2^) transepithelial CXL support is a safe and effective treatment of progressive keratoconus. After this treatment, postoperative epithelial thickness and pachymetry thickness initially decreased then returned to baseline levels at postoperative 3 months and achieved stabilization at postoperative 12 months. The stabilities of the corneal pachymetry and epithelial thickness can therefore be regarded as a parameter of clinical effectiveness. However, further clinical studies with longer follow-ups are necessary to fully assess the long-term efficacy of accelerated transepithelial CXL.

## Additional Information

**How to cite this article**: Zhang, X. *et al*. One-year Outcomes of Pachymetry and Epithelium Thicknesses after Accelerated (45mW/cm^2^) Transepithelial Corneal Collagen Cross-linking for Keratoconus Patients. *Sci. Rep.* **6**, 32692; doi: 10.1038/srep32692 (2016).

## Figures and Tables

**Table 1:** Preoperation Characteristics of Enrolled Keratoconus Patients.

Variable		Mean±SD	Range
Male		21	—
Female		7	—
OD		18	—
OS		10	—
Examination	Age (years)	23.61 ± 5.05	(10, 34)
	Sphere (D)	−5.17 ± 3.60	(−16.00, −0.50)
	Cylinder (D)	−2.51 ± 1.70	(−6.00, 0)
	Spherical Equivalent (D)	−6.23 ± 3.81	(−18.00, −0.63)
	UDVA (logMAR)	1.02 ± 0.55	(0.10, 2.00)
	CDVA (logMAR)	0.31 ± 0.35	(−0.18, 1.30)
	Intracular pressure (mm Hg)	12.29 ± 3.16	(7, 19.5)
	ECD (cells/mm^2^)	3264.67 ± 382.09	(2752, 3925)
	K_max_ (D)	56.18 ± 7.90	(43.4, 71.7)
	Astigmatism (D)	2.64 ± 1.90	(0.1, 5.9)
	Thinnest Point (μm)		
	OCT	443.18 ± 39.75	(374, 521)
	Rotating Scheimpflug tomography	459.46 ± 37.52	(393, 528)
	Corneal Anterior Surface Elevation	22.54 ± 14.28	(+4, +49)
	Corneal Posterior Surface Elevation	46.46 ± 27.09	(+6, +114)

D, diopters; UDVA, uncorrected distance visual acuity; CDVA, corrected distance visual acuity; logMAR, logarithm of the minimal angle of resolution; ECD, endothelial cell density; K_max_, maximum K value; OCT, optical coherence tomography.

**Table 2:** Mean values during 1-year’s follow-up.

Parameter	Preoperative (n = 28 eyes)	Postoperative				
		1 Week (n = 16 eyes) P*	1 Month (n = 21 eyes) P*	3 Months (n = 23 eyes) P*	6 Months (n = 25 eyes) P*	12 Months (n = 12 eyes) P*
UDVA (logMAR)	1.02 ± 0.55	0.78 ± 0.49	0.70 ± 0.49	0.79 ± 0.43	0.74 ± 0.41	0.58 ± 0.34
		(P = 0.119)	(P = 0.023)	(P = 0.243)	(P = 0.022)	(P=0.023)
CDVA (logMAR)	0.31 ± 0.35	0.28 ± 0.31	0.26 ± 0.25	0.20 ± 0.24	0.19 ± 0.18	0.15 ± 0.19
		(P = 0.205)	(P = 0.804)	(P = 0.236)	(P = 0.089)	(P = 0.056)
Spherical Equivalent (D)	−6.23 ± 3.81	−5.33 ± 3.36	−4.75 ± 2.60	−5.04 ± 2.97	−4.83 ± 2.91	−5.35 ± 3.41
		(P = 0.751)	(P = 0.258)	(P = 0.444)	(P = 0.087)	(P = 0.241)
Intracular pressure (mm Hg)	12.29 ± 3.16	14.70 ± 2.99	13.78 ± 2.11	11.05 ± 3.12	10.74 ± 3.46	10.68 ± 3.72
		(P = 0.305)	(P = 0.942)	(P = 0.409)	(P=0.050)	(P = 0.359)
ECD (cells/mm^2^)	3264.67 ± 382.09	3268.35 ± 371.55	3291.92 ± 366.84	3249.56 ± 386.30	3173.89 ± 467.56	3303.92 ± 309.67
		(P = 0.786)	(P = 0.540)	(P = 0.749)	(P = 0.968)	(P = 0.621)
K_max_ (D)	56.18 ± 7.90	54.56 ± 8.81	55.78 ± 8.11	56.37 ± 8.71	55.80 ± 7.92	55.47 ± 8.24
		(P = 0.053)	(P = 0.274)	(P = 0.821)	(P = 0.763)	(P = 0.916)
Astigmatism (D)	2.64 ± 1.90	2.57 ± 1.60	2.76 ± 2.11	2.88 ± 1.83	3.06 ± 2.09	2.74 ± 2.02
		(P = 0.973)	(P = 0.414)	(P = 0.549)	(P = 0.078)	(P = 0.829)
Thinnest point (μm)						
OCT	443.18 ± 39.75	447.21 ± 43.43	442.40 ± 40.83	441.16 ± 34.55	441.13 ± 37.10	439.04 ± 44.99
		(P = 0.019)	(P = 0.060)	(P = 0.781)	(P = 0.211)	(P = 0.109)
Rotating Scheimpflug tomography	459.46 ± 37.52	466.95 ± 41.24	459.76 ± 36.66	451.50 ± 33.68	460.46 ± 38.72	454.78 ± 44.48
		(P = 0.442)	(P = 0.769)	(P = 0.757)	(P = 0.878)	(P = 0.376)
Corneal Posterior Surface Elevation	44.35 ± 26.71	37.75 ± 26.55	42.81 ± 28.44	42.71 ± 28.15	45.17 ± 28.75	41.13 ± 28.49
		(P = 0.356)	(P = 0.048)	(P = 0.412)	(P = 0.919)	(P = 0.581)

UDVA, uncorrected distance visual acuity; CDVA, corrected distance visual acuity; logMAR, logarithm of the minimal angle of resolution; D, diopters; ECD, endothelial cell density; K_max_, maximum K value; OCT, optical coherence tomography.

^*^Compared with preoperative.

**Table 3:** Epithelium Thickness (μm) at each measuring point over time (Mean ± SD).

Sectoral Epithelial Thickness(mm)	Preoperative (n = 28 eyes)	Postoperative				
		1 Week (n = 19 eyes) P^*^	1 Month (n = 20 eyes) P^*^	3 Months (n = 19 eyes) P^*^	6 Months (n = 23 eyes) P^*^	12 Months (n = 23 eyes) P^*^
Min	41.68 ± 8.78	37.37 ± 11.09	43.90 ± 8.01	41.53 ± 9.32	42.70 ± 8.54	40.96 ± 10.27
		(P < 0.001)	(P = 0.895)	(P=0.779)	(P = 0.118)	(P = 0.183)
Max	65.57 ± 7.10	62.53 ± 7.74	64.25 ± 8.44	65.68 ± 8.88	64.96 ± 7.82	65.26±6.22
		(P = 0.089)	(P = 0.615)	(P = 0.877)	(P = 0.963)	(P = 0.656)
Min-Max	−24.21 ± 12.56	−25.05 ± 15.35	−20.40 ± 10.86	−24.16 ± 14.04	−23.00 ± 12.06	−24.30 ± 13.87
		(P = 0.025)	(P = 0.508)	(P = 0.940)	(P = 0.082)	(P = 0.350)
Center	50.39 ± 6.88	47.47 ± 6.64	50.95 ± 6.59	51.63 ± 5.56	50.22 ± 6.40	50.39 ± 7.58
		(P < 0.001)	(P = 0.770)	(P = 0.436)	(P=0.354)	(P = 0.894)
Paracenteral-S	58.07 ± 5.54	52.89 ± 6.60	57.45 ± 6.78	58.21 ± 6.36	57.48 ± 4.39	58.48 ± 4.48
		(P < 0.001)	(P = 0.735)	(P = 0.745)	(P = 0.859)	(P = 0.337)
Paracenteral-SN	58.75 ± 5.63	53.84 ± 4.07	57.90 ± 6.92	59.32 ± 6.52	59.00 ± 6.77	58.96 ± 4.62
		(P < 0.001)	(P = 0.410)	(P = 0.849)	(P = 0.268)	(P = 0.254)
Paracenteral-N	57.75 ± 6.58	52.84 ± 4.98	56.50 ± 5.84	58.05 ± 6.93	57.52 ± 6.19	57.83 ± 4.79
		(P < 0.001)	(P = 0.196)	(P = 0.866)	(P = 0.906)	(P = 0.266)
Paracenteral-IN	55.32 ± 4.97	50.32 ± 5.51	54.60 ± 7.56	55.37 ± 6.23	54.22 ± 4.94	54.04 ± 5.76
		(P < 0.001)	(P = 0.478)	(P = 0.953)	(P = 0.423)	(P=0.196)
Paracenteral-I	52.18 ± 7.62	47.37 ± 6.29	52.75 ± 7.17	51.42 ± 7.31	51.13 ± 6.39	51.04 ± 7.26
		(P < 0.001)	(P = 0.331)	(P = 0.877)	(P = 0.651)	(P = 0.255)
Paracenteral-IT	49.82 ± 7.48	46.47 ± 6.36	50.85 ± 7.77	48.89 ± 7.83	48.96 ± 6.30	47.91 ± 8.52
		(P < 0.001)	(P = 0.376)	(P = 0.819)	(P = 0.959)	(P = 0.049)
Paracenteral-T	51.25 ± 7.34	47.37 ± 6.95	51.65 ± 6.77	50.47 ± 6.62	51.83 ± 5.69	51.13 ± 8.08
		(P = 0.002)	(P = 0.422)	(P = 1.000)	(P = 0.061)	(P = 0.851)
Paracenteral-ST	56.89 ± 6.43	52.11 ± 7.31	56.60 ± 7.82	57.21 ± 6.67	57.52 ± 6.19	57.87 ± 6.12
		(P < 0.001)	(P = 0.836)	(P = 0.689)	(P = 0.069)	(P = 0.341)
Mid-peripheral-S	56.11 ± 5.63	51.58 ± 6.08	55.70 ± 6.47	55.11 ± 5.44	54.65 ± 3.94	55.74 ± 4.97
		(P = 0.001)	(P = 0.738)	(P = 0.863)	(P = 0.214)	(P = 0.829)
Mid-peripheral-SN	57.29 ± 5.09	52.84 ± 5.70	56.90 ± 6.54	57.11 ± 6.11	56.87 ± 6.33	57.09 ± 4.04
		(P < 0.001)	(P = 0.670)	(P = 0.956)	(P = 0.889)	(P = 0.410)
Mid-peripheral-N	59.71 ± 6.07	54.79 ± 4.08	57.70 ± 6.45	58.11 ± 5.29	59.61 ± 8.18	59.43 ± 4.00
		(P < 0.001)	(P = 0.086)	(P = 0.490)	(P = 0.677)	(P = 0.551)
Mid-peripheral-IN	60.50 ± 4.97	54.63 ± 4.72	58.45 ± 7.72	57.68 ± 4.42	59.04 ± 4.97	59.48 ± 5.11
		(P < 0.001)	(P = 0.312)	(P = 0.155)	(P = 0.210)	(P = 0.426)
Mid-peripheral-I	57.46 ± 7.41	52.47 ± 3.53	56.85 ± 4.99	55.26 ± 6.76	56.22 ± 5.85	57.09 ± 5.49
		(P < 0.001)	(P = 0.171)	(P = 0.982)	(P = 0.457)	(P = 0.497)
Mid-peripheral-IT	55.00 ± 6.45	50.58 ± 5.91	54.30 ± 6.59	52.58 ± 7.40	53.52 ± 7.04	52.61 ± 6.79
		(P = 0.001)	(P = 0.204)	(P = 0.832)	(P = 0.464)	(P = 0.008)
Mid-peripheral-T	56.07 ± 5.87	49.95 ± 7.06	55.35 ± 6.97	53.89 ± 6.27	57.04 ± 6.85	55.26 ± 6.56
		(P = 0.005)	(P = 0.510)	(P = 0.539)	(P = 0.176)	(P = 0.587)
Mid-peripheral-ST	58.21 ± 6.60	53.42 ± 7.18	57.30 ± 7.38	58.00 ± 7.94	57.65 ± 7.13	58.30 ± 5.37
		(P < 0.001)	(P = 0.909)	(P = 0.834)	(P = 0.930)	(P = 0.921)

**Table 4:** Pachymetry Thickness (μm) at each measuring point over time (Mean ± SD).

Sectoral Epithelial Thickness(mm)	Preoperative (n = 28 eyes)	Postoperative				
		1 Week (n = 19 eyes) P*	1 Month (n = 20 eyes) P*	3 Months (n = 19 eyes) P*	6 Months (n = 23 eyes) P*	12 Months (n = 23 eyes) P*
SN-IT (2–5 mm)	49.54 ± 28.66	44.58 ± 16.17	44.50 ± 21.76	53.68 ± 21.58	52.35 ± 26.47	51.26 ± 23.84
		(P = 0.921)	(P = 0.793)	(P = 0.927)	(P = 0.357)	(P = 0.092)
S-I (2–5 mm)	40.21 ± 27.35	35.89 ± 17.58	36.80 ± 24.26	44.26 ± 19.89	45.74 ± 23.39	44.65 ± 29.86
		(P = 0.741)	(P = 0.989)	(P = 0.805)	(P = 0.091)	(P = 0.121)
Min	443.18 ± 39.75	447.21 ± 43.43	442.40 ± 40.83	441.16 ± 34.55	441.13 ± 37.10	439.04 ± 44.99
		(P = 0.019)	(P = 0.060)	(P = 0.781)	(P = 0.211)	(P = 0.109)
Min-Median	−52.07 ± 21.20	−47.37 ± 21.78	−49.05 ± 24.17	−49.84 ± 19.97	−52.39 ± 21.25	−50.65 ± 24.02
		(P = 0.938)	(P = 0.686)	(P = 0.502)	(P = 0.764)	(P = 0.240)
Min-Max	−106.36 ± 34.01	−99.11 ± 34.56	−100.15 ± 34.47	−104.74 ± 33.34	−106.78 ± 33.88	−104.00 ± 38.47
		(P = 0.767)	(P = 0.775)	(P = 0.630)	(P = 0.981)	(P = 0.663)
Center	466.75 ± 38.54	470.79 ± 44.65	464.85 ± 35.87	463.05 ± 32.55	464.74 ± 36.69	461.74 ± 40.55
		(P = 0.353)	(P = 0.038)	(P = 0.962)	(P = 0.266)	(P = 0.510)
Paracenteral-S	526.71 ± 35.88	523.16 ± 33.39	520.15 ± 34.08	524.84 ± 33.38	526.83 ± 34.96	521.43 ± 33.66
		(P = 0.033)	(P = 0.061)	(P = 0.986)	(P = 0.851)	(P = 0.965)
Paracenteral-SN	524.00 ± 36.06	521.53 ± 32.52	519.55 ± 34.15	522.21 ± 32.61	523.39 ± 33.60	515.43 ± 37.48
		(P = 0.063)	(P = 0.119)	(P = 0.988)	(P = 0.844)	(P = 0.596)
Paracenteral-N	511.57 ± 35.26	510.63 ± 34.20	507.65 ± 34.17	507.47 ± 33.40	506.96 ± 33.23	506.48 ± 33.76
		(P = 0.087)	(P = 0.117)	(P = 0.861)	(P = 0.157)	(P = 0.900)
Paracenteral-IN	499.36 ± 33.66	500.26 ± 35.67	495.70 ± 34.60	493.42 ± 32.78	492.26 ± 32.79	490.78 ± 37.00
		(P = 0.093)	(P = 0.081)	(P = 0.771)	(P = 0.003)	(P = 0.088)
Paracenteral-I	486.50 ± 35.50	488.63 ± 36.13	483.35 ± 39.80	480.58 ± 33.71	481.09 ± 34.39	476.78 ± 40.98
		(P = 0.019)	(P = 0.082)	(P = 0.880)	(P = 0.011)	(P = 0.010)
Paracenteral-IT	474.46 ± 33.85	476.26 ± 35.58	475.10 ± 34.29	468.05 ± 31.52	472.39 ± 34.04	468.52 ± 35.31
		(P = 0.056)	(P = 0.325)	(P = 0.990)	(P = 0.449)	(P = 0.116)
Paracenteral-T	483.75 ± 32.96	483.58 ± 35.76	481.40 ± 33.04	478.95 ± 30.01	490.26 ± 37.37	481.35 ± 33.61
		(P = 0.127)	(P = 0.159)	(P = 0.953)	(P = 0.174)	(P = 0.821)
Paracenteral-ST	512.04 ± 33.86	509.74 ± 34.65	506.50 ± 35.06	509.47 ± 31.93	514.87 ± 34.80	509.52 ± 34.35
		(P = 0.124)	(P = 0.050)	(P = 0.961)	(P = 0.136)	(P = 0.558)
Mid-peripheral-S	559.54 ± 39.24	554.58 ± 33.19	549.90 ± 34.73	555.84 ± 33.03	558.17 ± 33.39	552.96 ± 33.42
		(P = 0.191)	(P = 0.084)	(P = 0.938)	(P = 0.736)	(P = 0.766)
Mid-peripheral-SN	556.96 ± 38.26	553.00 ± 32.16	551.25 ± 34.08	553.42 ± 31.63	553.74 ± 32.36	551.00 ± 32.99
		(P = 0.217)	(P = 0.282)	(P = 0.869)	(P = 0.559)	(P = 0.959)
Mid-peripheral-N	546.43 ± 36.06	543.68 ± 32.09	542.55 ± 36.44	540.95 ± 32.68	542.87 ± 32.84	541.57 ± 34.13
		(P = 0.084)	(P = 0.175)	(P = 0.766)	(P = 0.211)	(P = 0.841)
Mid-peripheral-IN	542.29 ± 34.91	537.68 ± 34.57	534.70 ± 35.98	533.89 ± 33.50	534.35 ± 33.66	532.74 ± 36.38
		(P = 0.080)	(P = 0.040)	(P = 0.646)	(P = 0.001)	(P = 0.160)
Mid-peripheral-I	533.79 ± 35.62	530.95 ± 35.89	519.15 ± 44.30	527.21 ± 336.48	527.35 ± 35.31	524.35 ± 40.55
		(P = 0.134)	(P = 0.016)	(P = 0.939)	(P = 0.001)	(P = 0.089)
Mid-peripheral-IT	518.39 ± 32.23	514.95 ± 35.52	513.10 ± 36.55	512.89 ± 37.16	514.43 ± 34.83	509.26 ± 34.17
		(P = 0.039)	(P = 0.081)	(P = 0.977)	(P = 0.257)	(P = 0.029)
Mid-peripheral-T	518.82 ± 30.49	515.63 ± 31.43	513.65 ± 34.42	512.79 ± 32.01	521.83 ± 35.13	515.39 ± 31.93
		(P = 0.230)	(P = 0.159)	(P = 0.888)	(P = 0.282)	(P = 0.854)
Mid-peripheral-ST	545.96 ± 34.16	542.11 ± 33.00	537.60 ± 35.39	543.00 ± 33.56	548.87 ± 35.22	542.65 ± 33.08
		(P = 0.311)	(P = 0.041)	(P = 0.997)	(P = 0.292)	(P = 0.585)

S, superior; N, nasal; I, inferior; T, temporal.

^*^Compared with preoperative.
